# Identification of *Bradyrhizobium elkanii* Genes Involved in Incompatibility with *Vigna radiata*

**DOI:** 10.3390/genes8120374

**Published:** 2017-12-08

**Authors:** Hien P. Nguyen, Hiroki Miwa, Takakazu Kaneko, Shusei Sato, Shin Okazaki

**Affiliations:** 1 Graduate School of Agriculture, Tokyo University of Agriculture and Technology, Saiwai-cho, Fuchu City, Tokyo 183-0054, Japan; nguyenphuochien92@gmail.com (H.P.N.); miwahiroki@hotmail.com (H.M.); 2 Faculty of Life Sciences, Kyoto Sangyo University, Motoyama, Kamigamo, Kita-Ku, Kyoto 603-8555, Japan; tkaneko@cc.kyoto-su.ac.jp; 3 Graduate School of Life Sciences, Tohoku University, Katahira, Aoba-ku, Sendai, Miyagi 980-8577, Japan; shuseis@ige.tohoku.ac.jp

**Keywords:** symbiosis, nodulation, *Bradyrhizobium elkanii*, *Vigna radiata*, *Rj4* soybean

## Abstract

The establishment of a root nodule symbiosis between a leguminous plant and a rhizobium requires complex molecular interactions between the two partners. Compatible interactions lead to the formation of nitrogen-fixing nodules, however, some legumes exhibit incompatibility with specific rhizobial strains and restrict nodulation by the strains. *Bradyrhizobium elkanii* USDA61 is incompatible with mung bean (*Vigna radiata* cv. KPS1) and soybean cultivars carrying the *Rj4* allele. Here, we explored genetic loci in USDA61 that determine incompatibility with *V. radiata* KPS1. We identified five novel *B. elkanii* genes that contribute to this incompatibility. Four of these genes also control incompatibility with soybean cultivars carrying the *Rj4* allele, suggesting that a common mechanism underlies nodulation restriction in both legumes. The fifth gene encodes a hypothetical protein that contains a *tts* box in its promoter region. The *tts* box is conserved in genes encoding the type III secretion system (T3SS), which is known for its delivery of virulence effectors by pathogenic bacteria. These findings revealed both common and unique genes that are involved in the incompatibility of *B. elkanii* with mung bean and soybean. Of particular interest is the novel T3SS-related gene, which causes incompatibility specifically with mung bean cv. KPS1.

## 1. Introduction

Symbiotic relationships between leguminous plants and soil bacteria, collectively termed rhizobia, are characterized by the formation of root nodules, which are specialized plant organs that accommodate the rhizobia. In the root nodules, rhizobia fix atmospheric dinitrogen into ammonia, which is utilized by the host legumes. This symbiotic association is of critical importance in agricultural production and the nitrogen cycle. The interactions between legumes and rhizobia exhibit high levels of specificity, which is determined by the exchange of signal molecules. Legume roots excrete flavonoid compounds that interact specifically with the transcriptional regulator NodD in rhizobia [1,2]. The activation of NodD induces the expression of nodulation genes, which are required for the synthesis of lipo-chitooligosaccharides called Nod-factors [1,2]. The Nod factors function as signals for host receptors, which activate signal pathways leading to rhizobial infection and nodule formation [1,2]. In addition, rhizobial factors, such as exopolysaccharides, lipopolysaccharides, and secreted proteins, have also been reported to affect nodulation and host specificity [3,4].

The rhizobium *Bradyrhizobium elkanii* forms symbiotic relationships with a number of legumes, including soybean, mung bean (*Vigna radiata*), and groundnuts (*Arachys hypogaea*). In Brazil, *B. elkanii* is used as an inoculant for soybean cultivation [5]. The formation of symbiotic root nodules in soybean is controlled by several loci, which are referred to as the *rj* or *Rj* loci [6]. Soybean varieties carrying the dominant *Rj4* allele, such as the cultivars Hill and BARC2, are incapable of nodulation with *B. elkanii* USDA61, but are compatible with *Bradyrhizobium diazoefficiens* USDA110 [7]. These studies revealed that *Rj4* incompatibility is strain-specific and cultivar-specific, and appears to resemble gene-for-gene resistance in plant–pathogen interactions [8]. The recent identification of genes that are encoded by the *Rj2* and *Rj4* genes supports this idea. *Rj2* encodes a member of the Toll-interleukin receptor/nucleotide binding site/leucine-rich repeat (TIR-NBS-LRR) class of proteins [9], while *Rj4* encodes a thaumatin-like protein [10]. Both of these classes of protein are involved in plant resistance to pathogens.

Mung bean is an important leguminous crop in tropical and subtropical regions. Growth and seed production by *V. radiata* are highly dependent on symbiosis with nitrogen-fixing rhizobia [11]. The *V. radiata* cv. KPS1 cultivar is incompatible with *B. elkanii* USDA61, resulting in ineffective nodulation [12]. In a previous study [12], we mutated two *B. elkanii* genes, *rhcC2* and *rhcJ*, which are well-conserved components of the type III secretion system (T3SS). The T3SS is a complex protein transport system that is involved in plant and animal pathogenesis by Gram-negative bacteria, and it is also found in many rhizobia [13,14,15,16,17]. The *B. elkanii* T3SS mutants formed functional nodules on *V. radiata* cv. KPS1 and *Glycine max* cv. Hill plants [12]. These results implicated the T3SS in the control of incompatibility between rhizobia and their plant hosts, however, genetic elements involved in the incompatibility between *B. elkanii* USDA61 and *V. radiata* cv. KPS1 have not been fully elucidated. Here, we identified five novel *B. elkanii* genes that contribute to this incompatibility. Four of these genes also control nodulation restriction in *Rj4* soybean, suggesting a common mechanism that is underlying both incompatible interactions. The fifth gene encodes a T3SS-related protein with a host-specific function in KPS1. These findings shed light on the diverse and complicated mechanisms underlying the incompatible symbiosis, and suggest the involvement of a novel T3SS-related gene in the nodulation restriction of mung bean.

## 2. Materials and Methods 

### 2.1. Bacterial Strains and Growth Conditions

The bacterial strains and plasmids that were used in this study are listed in Table 1. *B. elkanii* strains were grown at 28 °C in arabinose–gluconate medium [18], supplemented with 50 μg mL^−1^ polymyxin and 200 μg mL^−1^ kanamycin. *Escherichia coli* strains were grown at 37 °C in Luria-Bertani medium (LB) [19] supplemented with 50 μg mL^−1^ kanamycin.

### 2.2. Transposon Mutagenesis of B. elkanii USDA61 

The transposon mini-Tn*5* was introduced into the *B. elkanii* USDA61 strain by conjugation, as described by Krause et al. [23], using *E. coli* strain S17-1 [20] carrying the plasmid pUTKm [21], and *E. coli* HB101 (pRK2013) [22]. Strains containing transposons were selected on arabinose–gluconate agar plates, supplemented with 50 μg mL^−1^ polymyxin and 200 μg mL^−1^ kanamycin.

### 2.3. Plant Cultivation, Mutant Screening, and Inoculation Tests 

Seeds of *V. radiata* cv. KPS1 and *G. max* cv. BARC2 were surface sterilized and germinated on wet sterile paper, then the seedlings were transferred to sterile vermiculite, using procedures described previously [8]. One day after transplantation, 216 *V. radiata* KPS1 seedlings were inoculated with approximately 36,000 Tn*5* insertion strains of *B. elkanii* USDA61 (1 mL of 10^7^ cells per mL on each plant). Plants were cultivated in a plant growth cabinet (LPH-410SP; NK Systems Co. Ltd., Osaka, Japan) at 25 °C and 70% humidity under a day/night regimen of 16/8 h. After 30 days of cultivation, nodules that formed on the *V. radiata* KPS1 plants were sampled, and the *B. elkanii* mutants were isolated, as described previously [8]. These isolates were used to re-inoculate *V. radiata* KPS1 plants in order to confirm the nodulation phenotypes. *G. max* cv. BARC2 seedlings were also inoculated with the identified mutants in order to determine if the mutants could form nodules on this *G. max* cultivar. Inoculation tests were performed, as described previously [8].

### 2.4. Nucleotide Sequence Analysis of Tn5-Flanking Regions 

Genomic DNA was isolated from *B. elkanii* strains using the Wizard Genomic DNA Purification Kit (Promega, Madison, WI, USA). Transposon-flanking sequences were amplified using the Y-linker method [24]. The genomic DNA was digested with NlaIII or SphI, and ligated with the Y-linker. PCR was carried out using the GoTaq Green Master Mix (Promega), Y-linker primer and Tn*5* primer (Table 1). The thermal cycling conditions were as described previously [8]. The PCR fragments were sequenced directly using the Tn*5* or Y-linker primers. Homology searches were carried out using BLASTN at the National Center for Biotechnology Information and an in-house database for the *B. elkanii* USDA61 genome [25]. For the identification of *tts* box (TCGTCAG[CGT]TTNTCG[TA][CA]AGCTN(8)[TC]A) motif, the 2 kb upstream sequences of each gene were searched with the program “fuzznuc” of the EMBOSS package [26]. The consensus sequences from *Rhizobium* sp. strain NGR234 [27] and *B. diazoefficiens* USDA110 [23] were compared to select the *tts* box motif sequence (data not shown). In order to confirm that the observed phenotypes of the mutants were caused by transposon insertion and not by a secondary mutation independent of the transposon, *innB* identified in BE53 was mutated via single-crossover recombination. The internal region of *innB* was amplified by PCR using the primers InnBXbaI-intF and InnBEcoRI-intR (Table 1). The PCR product was cloned into the vector pSUPSCAKm and was mobilized into *B. elkanii* USDA61 via conjugation, as described previously [12]. The mutant generated by integration of the plasmids was selected on PSY medium containing polymyxin and kanamycin. Integration of the plasmids was confirmed by PCR using the primer set Psuppol-F and Psuppol-R (Table 1).

### 2.5. Microscopy 

Nodules were excised from *V. radiata* cv. KPS1 and *G. max* cv. BARC2 and were fixed, sectioned, and photographed under a stereoscopic microscope, as described previously [8].

### 2.6. Nucleotide Sequence Accession Numbers 

The sequences of the *innA*, *innB*, *innC*, *innD*, and *innE* genes have been submitted to GenBank and may be found under accession numbers KX499540, KX499541, KX499542, KX499543, and KX499544, respectively.

## 3. Results

### 3.1. Isolation of the Transposon Mutants of B. elkanii

**Figure 1:**
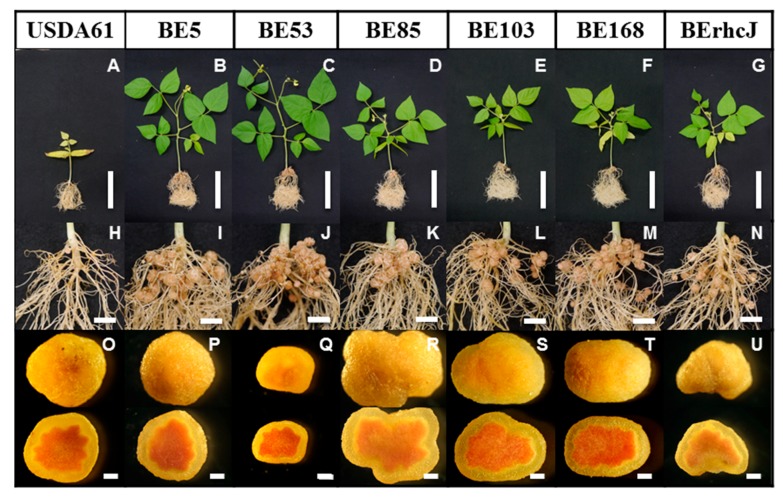
*Vigna radiata* (L.) cv. KPS1 plants, roots, and nodules inoculated with *Bradyrhizobium elkanii* USDA61, Tn*5* mutants and type III secretion system-deficient mutant BErhcJ. Plants were photographed at 40 days post-inoculation. Scale bars: (**A**–**G**) 10 cm, (**H**–**N**) 1 cm, and (**O**–**U**) 1 mm.

To investigate the molecular mechanisms underlying the incompatibility of *V. radiata* cv. KPS1 with *B. elkanii* USDA61, we created about 36,000 Tn*5* transposon insertion mutants of USDA61, and then inoculated 216 KPS1 seedlings with the mutants. Tn*5* mutants that formed nodules on KPS1 were isolated and used to re-inoculate KPS1 plants to confirm the nodulation phenotypes. Five mutants (BE5, BE53, BE85, BE103, and BE168) were confirmed to form nodules on KPS1 (Figure 1). The wild-type strain USDA61 formed only a small number of nodules on KPS1 plants, and these plants exhibited nitrogen deficiency symptoms (Figure 1A,H). In contrast, all of the Tn*5* mutants were able to form mature nodules and promote the growth of KPS1, indicating efficient nitrogen-fixing activity (Figure 1B–F,I–M). The *B. elkanii* strain BErhcJ, a mutant defective in the type III protein secretion system [12], also formed nodules and promoted growth of KPS1 plants (Figure 1G,N). Nodules formed by all the plants showed a red color in the central tissue due to the presence of leghemoglobin, which is an indicator of nitrogenase activity (Figure 1O–U).

### 3.2. Symbiotic Phenotypes of the Transposon Mutants on V. radiata cv. KPS1 Plants

**Figure 2:**
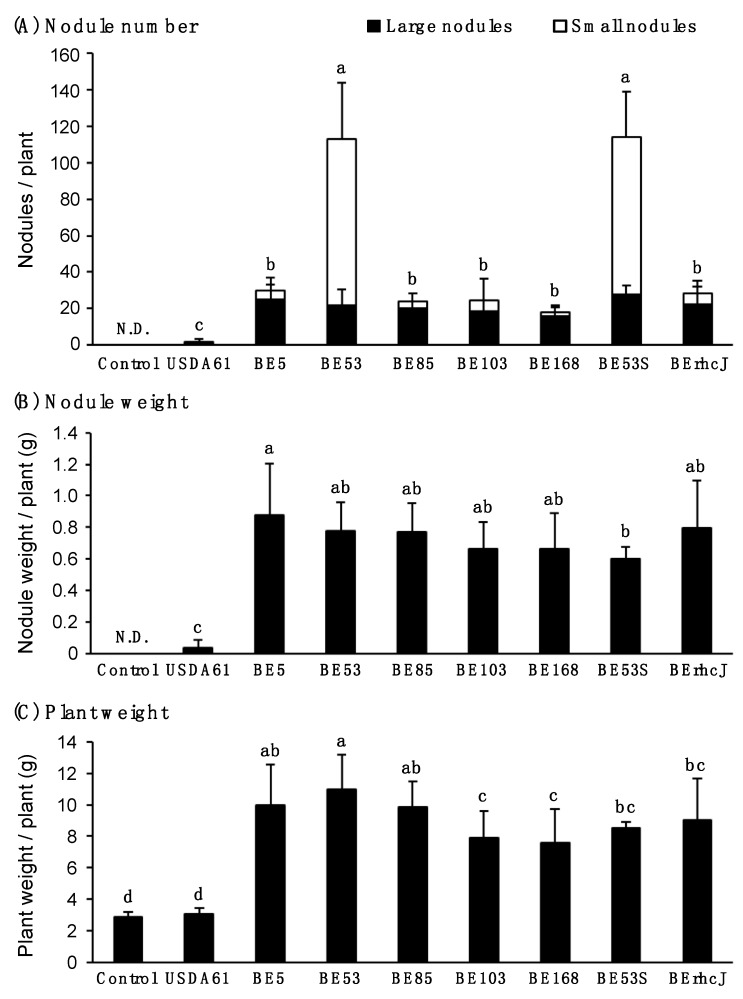
Symbiotic properties of *V. radiata* (L.) cv. KPS1 inoculated with *B. elkanii* USDA61, Tn*5* mutants and type III secretion system-deficient mutant BErhcJ. (**A**) Nodule number, (**B**) nodule weight and (**C**) plant weight measured at 40 days post inoculation. Open bars and closed bars in (**A**) show the numbers of small nodules (<2 mm) and large nodules (≥2 mm), respectively. Nodulation tests were performed at least five times, the values are means of seven mung bean plants, and the error bars indicate standard deviations. Statistical analysis (Fisher’s method) was performed comparing the total nodule numbers induced by USDA61, BErhcJ and Tn*5* mutants. Means followed by the same letters are not significantly different at 5% level of significance by Fisher’s test. N.D.: Not determined.

To characterize the symbiotic phenotypes of the mutants, we measured the nodule numbers, nodule weights, and plant fresh weights of inoculated KPS1 plants at 40 days after inoculation (Figure 2). For this analysis, we classified the nodules into two types by size: <2 mm and ≥2 mm in diameter. The Tn*5* mutants exhibited significantly higher nodule numbers (about 18 to 114 nodules per plant) than the wild-type USDA61 strain (1.8 nodules per plant) (Figure 2A). The number of nodules and the total weight of nodules per plant were highest in plants that were inoculated with BE53 (Figure 2A,B). These plants also showed the highest fresh weights. Interestingly, BE53 induced many small nodules: 80% of total nodules (Figure 2A). In contrast, the mutants BE5, BE85, BE103, and BE168 formed mainly large nodules, and the numbers of large nodules and nodule weights per plant were similar to those of BErhcJ (Figure 2A,B). Apart from plants that were inoculated with BE53, the fresh weights of plants inoculated with the other mutants were similar to one another, and correlated with the numbers of large nodules and total nodule weights. These correlations were probably due to similar nitrogen fixation levels. As we reported previously, BErhcJ formed large nodules on KPS1 plants and promoted plant growth (Figure 2C). 

### 3.3. Symbiotic Phenotypes of the KPS1-Nodulating Mutants on Rj4 Soybean

**Figure 3:**
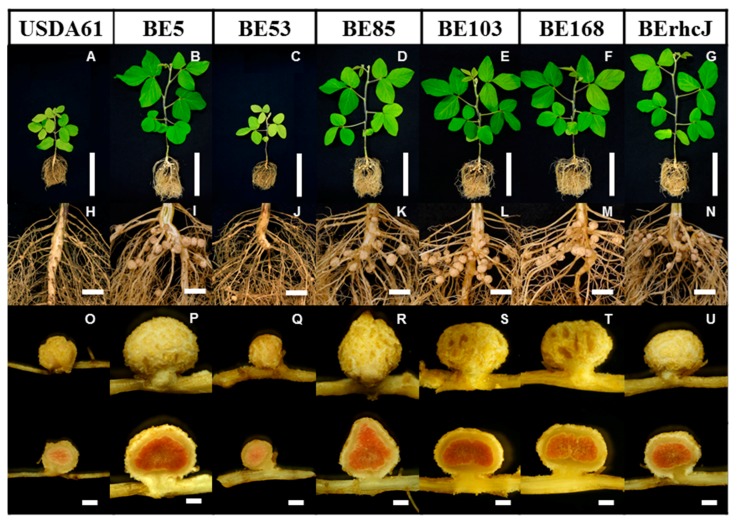
*Glycine max* cv. BARC2 (*Rj4Rj4*) plants, roots, and nodules inoculated with wild-type *B. elkanii* USDA61, Tn*5* mutants and type III secretion system-deficient mutant BErhcJ. Plants were photographed at 30 days after inoculation. Scale bars: (**A**–**G**) 10 cm, (**H**–**N**) 1 cm, and (**O**–**U**) 1 mm.

**Figure 4:**
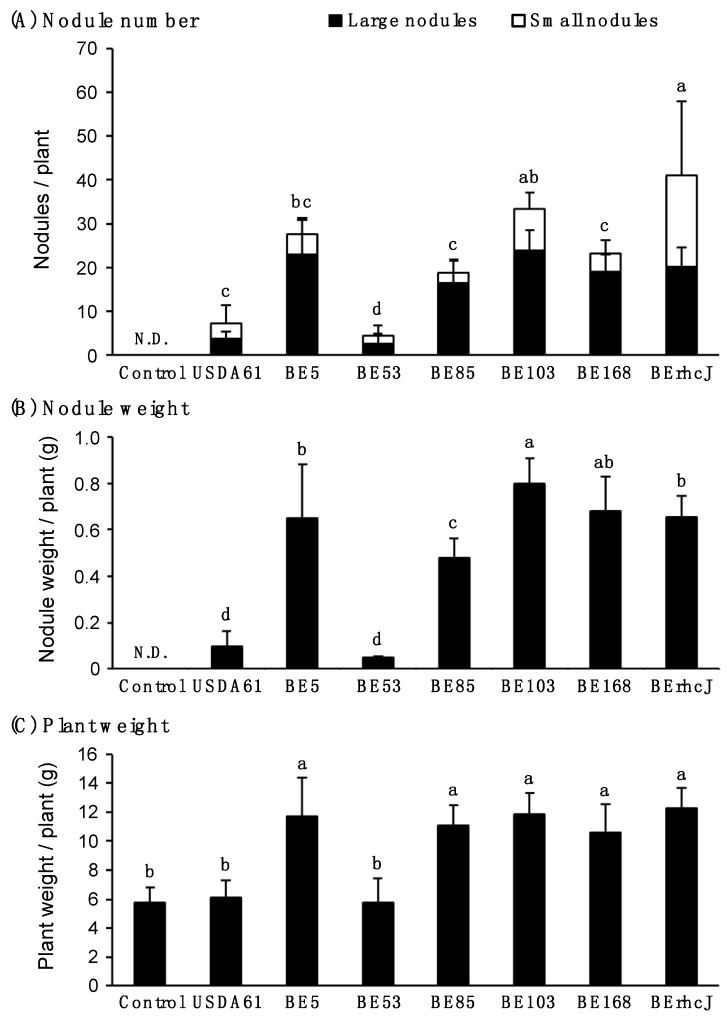
Symbiotic properties of *G. max* cv. BARC2 (*Rj4Rj4*) inoculated with *B. elkanii* USDA61, Tn*5* mutants and type III secretion system-deficient mutant BErhcJ. (**A**) Nodule number, (**B**) nodule weight and (**C**) plant weight measured at 30 days post inoculation. Open bars and closed bars in (**A**) show the numbers of small nodules (<2 mm) and large nodules (≥2 mm), respectively. Nodulation tests were performed at least two times, the values are means of six soybean plants, and the error bars indicate standard deviations. Statistical analysis (Fisher’s method) was performed comparing the total nodule numbers induced by USDA61, BErhcJ and Tn*5* mutants. Means followed by the same letters are not significantly different at 5% level of significance by Fisher’s test. N.D.: Not determined.

We also tested the symbiotic phenotypes of the Tn*5* mutants on soybean cultivar BARC2 (*Rj4*/*Rj4*), which is known to restrict nodulation by *B. elkanii* [8,12]. Wild-type USDA61 induced only a few nodules on BARC2 plants, and the plants exhibited retarded growth due to nitrogen deficiency (Figure 3A,H). Interestingly, the four mutants BE5, BE85, BE103, and BE168 induced numerous nodules on the BARC2 roots (Figure 3B,I,D–F,K–M). More than 75% of the nodules that were formed by these mutants were large (Figure 4A,B), and they enhanced plant growth (Figure 4C). In contrast, BE53, which formed numerous nodules on *V. radiata* KPS1, formed only a few nodules on soybean BARC2 (Figure 3C,J). The nodules formed by BE53 were small and irregular in shape, and were similar to those that were formed by USDA61 (Figure 3Q). The BARC2 plants inoculated with BE53 showed nitrogen deficiency symptoms, indicating that the small nodules were ineffective. 

### 3.4. B. elkanii Genes Responsible for the Symbiotic Incompatibility

To identify the genes that were disrupted by the Tn*5* insertions, Y-linker PCR was performed using genomic DNA from the Tn*5* mutants. All of the mutant genomes contain the Tn*5* fragment, and the PCR fragment sizes differ among strains, indicating that they are independent mutations. The genome of *B. elkanii* USDA61 has been completely sequenced [25], and the Tn*5* flanking regions all contain sequences that are found in the genome (Table 2). The location map and genome context of the identified genes indicated that these genes are located outside T3SS gene cluster (Figures S1 and S2). BE5 contains a Tn*5* insertion in a 1212 bp open reading frame (ORF), designated as *innA*, that encodes a cytosine deaminase, which is similar to those in rhizobia and other Gram-negative bacteria. BE85 contains a Tn*5* insertion in a 504 bp ORF (designated as *innC*) that encodes a tellurite resistance protein (TerB). The gene products are similar with those in other rhizobia and pathogenic bacteria. BE103 has an insertion in a 1089 bp ORF (*innD*) that encodes an ABC-transporter substrate-binding protein, whose homologues were well conserved among rhizobia as well as species of *Afipia*, *Rhodopseudomonas*, *Azospirillum*, and *Agrobacterium*. One of its homologues in *B. diazoefficiens* USDA110, blr3743, has been reported as a secreted protein [28]. BE168 has an insertion in a 2292 bp ORF (*innE*) encoding GTP pyrophosphokinase (Table 2). The *innE* product has 88% and 60% identity with the products of *relA* genes in rhizobia and *spoT* genes in other bacteria including *Rhodopseudomonas palustris*, *Xanthomonas campestris*, *Pseudomonas fluorescens*, and *Salmonella enterica*.

BE53 mutant carries a Tn*5* insertion in the 2280 bp ORF (*innB*), which encodes a hypothetical protein that is conserved among rhizobia as well as *Xanthomonas* and *Rhodopseudomonas*. Notably, a *tts* box motif (5′-TATGGGACCTAGCTTTCGAAAAGCTGACGA-3′) is present at 96 bp upstream of the *innB* ORF. The *tts* box motifs are often located in the promoter regions of genes encoding structural components of the T3SS and secreted proteins. Furthermore, a homologue of *innB* in *Mesorhizobium loti*, mlr6327 (GenBank accession No. BAB52639.1), is located in the cluster of genes that are encoding the T3SS [29].

In order to confirm that the observed phenotypes of the mutants were caused by transposon insertion and not by a secondary mutation independent of the transposon, we reintroduced the mutation into the wild-type background. The *innB* gene was mutated via single-crossover recombination in wildtype USDA61 and the newly constructed mutant, designated BE53S, was used to inoculate *V. radiata* cv. KPS1 plants, and its symbiotic phenotypes were examined. As a result, the BE53S formed numerous mature nodules similar to the transposon mutant (BE53) (Figure 2A–C), confirming that transposon insertion in BE53 was responsible for the altered nodulation phenotype.

## 4. Discussion

In fields, the application of high nitrogen-fixing rhizobia to their target legume crops often fails to increase the crop yields. One of the major causes of this phenomenon is that more competitive indigenous rhizobial strains, with low nitrogen fixation activity, occupy most of the nodules. This problem is known as the competition problem, and it is very important in the cultivation of legumes. Besides the competitiveness of different rhizobial strains, compatibility between leguminous plants and rhizobia also affect nodule occupancy. In soybean, *Rj* genes control nodulation: plants carrying specific *Rj* genes cannot form nodules when inoculated with specific rhizobial strains [6]. This compatibility/incompatibility might be reminiscent of host–pathogen interactions. Consistent with this observation, the *Rj2* and *Rj4* genes encode TIR-NBS-LRR and thaumatin-like proteins, respectively [10], and these proteins are involved in plant resistance to pathogens. 

Similarly, host-controlled nodulation occurs in mung beans, and the compatibility is dependent on both the host cultivar and the rhizobial strain. In a previous report, we showed that two genes that encode components of the T3SS, *rhcC2* and *rhcJ*, are necessary for the incompatibility between *V. radiata* cv. KPS1 and *B. elkanii* USDA61 [12]. The T3SS is involved in plant and animal pathogenesis, and many rhizobial strains possess a functional T3SS [13,14,15,16,17]. The secreted proteins, called nodulation outer proteins (Nops), affect symbiosis positively or negatively, depending on the host plant [23,30,31]. The expression of the rhizobial T3SS is controlled by flavonoids that are derived from the host legume. Flavonoids perceived by the NodD protein activate the expression of *ttsI*, a transcriptional activator of the type III secretion gene cluster (*ttsI*). TtsI subsequently activates the *ttsI* gene cluster, and proteins are secreted via the T3SS. A conserved motif, called the *tts* box, is located in the promoter region of genes encoding structural components and secreted effector proteins [23,32,33].

In the present study, we explored additional genetic determinants of *B. elkanii,* which are involved in the nodulation incompatibility between *V. radiata* KPS1 and *B. elkanii* USDA61. We performed Tn*5* transposon mutagenesis and identified five Tn*5* insertion mutants of *B. elkanii* USDA61, which were able to form functional nodules on *V. radiata* KPS1 and enhance the growth of the plants. 

The BE5 mutant contains a Tn*5* insertion in the *innA* gene, which encodes cytosine deaminase. Cytosine deaminase deaminates cytosine to form uracil and ammonia [34]. Although the mechanism for the involvement of cytosine deaminase in nodulation restriction remains unclear, the BE5 mutant efficiently nodulated and promoted the growth of both KPS1 and *Rj4* plants. The disruption of *innA* might cause the inactivation of the T3SS, which abolishes the secretion of effector proteins that are responsible for nodulation restriction in both *Rj4* soybean and *V. radiata* KPS1.

The BE53 mutant carries transposon in the *innB* gene, which encodes a hypothetical protein with an unknown function. The *innB* gene is preceded by the consensus *tts* box, and the *innB* homologue in *M. loti*, *mlr6327*, occurs in the cluster of genes encoding the T3SS [28]. Intriguingly, the *innB*-deficient mutant formed nodules on *V. radiata* KPS1 plants, but not on *Rj4* soybean. This suggests that *innB* encodes a protein with a host-specific function in KPS1. One possibility is that *innB* encodes an effector protein that is recognized specifically in *V. radiata* KPS1. It is likely that the rhizobial effector proteins that are recognized by KPS1 and *Rj4* soybean differ. The InnB might be recognized specifically by a resistance (R) protein in KPS1, causing nodulation restriction in KPS1, but not in *Rj4* soybean.

The BE85 mutant contains a Tn*5* insertion in *innC*, encoding TerB family tellurite resistance protein that is conserved among the *Bradyrhizobiaceae*. It has been reported that the Tellurium resistance proteins of *Frankia* spp. are induced by seed phenolic compounds that are produced by their actinorhizal host plants [35]. Considering that the disruption of *innC* induced nodulation on both KPS1 and *Rj4* plants, the gene might be involved in the T3SS. However, the function of these proteins in rhizobial symbiosis remains unknown.

The BE103 mutant carries a Tn*5* insertion in the gene *innD*, which encodes a substrate-binding protein in the ABC transporter family. These substrate-binding proteins are located in the periplasm, and are involved in the active transport of substrates into the cytoplasm [36,37,38]. Although the function of the *innD* protein remains unclear, its homologue in *B. diazoefficiens* USDA110 (*blr3743*, BAC49008.1) is secreted [28], and is upregulated in bacteroids in nodules [39,40]. Clearly, functional analyses are needed to determine the contribution of these transporters to the compatibility/incompatibility between rhizobia and their hosts. Since the inactivation of the *innD* gene by the Tn*5* insertion canceled the nodulation restriction in both KPS1 and *Rj4* plants, the *innD* product might be involved in the T3SS. Further molecular experiments will be performed to explore this possibility.

The BE168 mutant contains a Tn*5* insertion in *innE*, which encodes a GTP pyrophosphokinase (ppGpp synthetase) that is conserved among various rhizobia and pathogenic bacteria. *innE* shares similarity with the *relA* genes of *B. diazoefficens* USDA110 (BAC50330.1) and other bacteria. It has been proposed that ppGpp synthetase binds to, and alters the ability of, RNA polymerase to initiate and elongate transcription. ppGpp synthetase activates genes that function in the biosynthesis of amino acids and transcription factors that are responsible for stress responses [41,42]. In addition, ppGpp synthetase plays important roles in the virulence and the persistence of phytopathogens within host plants [43]. In *Rhizobium etli*, the *relA* gene regulates gene expression in bacteroid cells [44]. A *relA* mutant of *Sinorhizobium meliloti* shows a non-nodulation phenotype on alfalfa [45]. These findings suggest that the mutation in *innE* affects cellular ppGpp synthetase levels and leads to changes in cell functions that affect the strain specific incompatibility between *B. elkanii* USDA61 and mung bean and soybean. It should be noted that, except for the *innB* gene, which was confirmed by the newly constructed mutant BE53S, the other genes also need to be confirmed by mutational and/or complementation analysis to unequivocally associate the gene with the phenotype.

In addition to the genes and gene products discussed above, rhizobial factors have been reported to be involved in incompatible symbioses, including the type IV secretion system [46], extracellular polysaccharides [47], and peptidase that cleaves host-derived signaling peptides [48]. T3SSs in other rhizobia involved in incompatible symbioses have been reported for the following interactions: *Rhizobium* sp. NGR234 with *Crotalaria juncea* [49], *M. loti* MAFF303099 with *Lotus halophilus* [28], *Sinorhizobium fredii* USDA257 with *G. max* cv. McCall [15], *B. elkanii* USDA61 with *Rj4* soybean [8], *Bradyrhizobium japonicum* USDA122 with *Rj2* soybean [50], and *B. diazoefficiens* USDA110 with *V. radiata* KPS2 [51]. The type III-secreted proteins NopE1 and NopE2 of *B. diazoefficiens* USDA110 were reported as negative effectors of compatibility with *V. radiata* KPS2 [51]. In our preliminary results, *B. diazoefficiens* USDA110 formed nodules on *V. radiata* KPS1 and promoted plant growth efficiently (data not shown), indicating that NopE1 and NopE2 do not control incompatibility with KPS1, and that *B. elkanii* might possess other effectors that are recognized by KPS1. These findings suggest that the molecular mechanisms that are controlling incompatibility between various rhizobia and legumes differ, although the T3SS appears to underlie at least three types of nodulation restriction. Clearly, further study is necessary to define the roles of the T3SS components in both innate-immunity and rhizobial nodulation.

## 5. Concluding Remarks

In conclusion, we have identified novel genes of *B. elkanii* that are involved in incompatibility with *V. radiata* cv. KPS1. Four of the genes (*innA*, *innC*, *innD*, and *innE*) are also involved in incompatibility with *Rj4* soybean, suggesting that they participate in a common mechanism underlying nodulation restriction in both of the legumes. In contrast, *innB* is involved in nodulation restriction with *V. radiata* cv. KPS1, but not with *Rj4* soybean. The *innB* gene is preceded by a *tts* box, which is a promoter element in type III secretion system-related genes, suggesting its T3SS-related function. These findings shed light on the diverse and complicated mechanisms underlying the incompatible symbiosis, and suggest the involvement of a novel T3SS-related gene in the nodulation restriction of mung bean. 

## Figures and Tables

**Table 1:** Bacterial strains, plasmids and oligonucleotides used in this study.

Strain, Plasmid or Oligonucleotide	Characteristics or Sequence ^a^	Reference or Source
**Bacterial strains**		
*Bradyrhizobium elkanii*		
USDA61	Wild-type strain, Pol^r^	Keyser ^b^
BErhcJ	USDA61 derivative harboring insertion in *rhcJ* region, Pol^r^, Km^r^, Tc^r^	[12]
BE5	Tn*5* mutant of USDA61, Pol^r^, Km^r^	This study
BE53	Tn*5* mutant of USDA61, Pol^r^, Km^r^	This study
BE85	Tn*5* mutant of USDA61, Pol^r^, Km^r^	This study
BE103	Tn*5* mutant of USDA61, Pol^r^, Km^r^	This study
BE168	Tn*5* mutant of USDA61, Pol^r^, Km^r^	This study
BE53S	USDA61 derivative containing an insertion of plasmid pSUPSCAKm:int *innB*, Pol^r^, Km^r^	This study
*Escherichia coli*		
HB101	*recA*, *hsdR*, *hsdM*, *pro*, Sm^r^	Invitrogen, Carlsbad, CA, USA
S17-1	*pro recA* RP4-2(Tc^s^:Mu) (Km^s^:Tn*7*); Mob^+^	[20]
DH10B	Cloning strain	Invitrogen
**Plasmids**		
pUTKm	Transposon delivery vector; Ap^r^, Km^r^	[21]
pRK2013	Helper plasmid, ColE1 replicon carrying RK2 transfer genes; Km^r^, *tra*	[22]
pSUPSCAKm	Derivative of pSUPPOL2SCA [23] with a kanamycin resistance gene in the *DraI* site, *oriT* of RP4, Tc^r^, Km^r^	This study
pSUPSCAKm:int *innB*	pSUPSCAKm carrying a 0.5-kb DNA fragment containing internal sequence of *innB*, Km^r^	This study
**Oligonucleotides**		
Linker 1	5′-TTTCTGCTCGAATTCAAGCTTCTAACGATGTACGGGGACACATG-3′	[24]
Linker 2	5′-TGTCCCCGTACATCGTTAGAACTACTCGTACCATCCACAT-3′	[24]
Y-linker primer	5′-CTGCTCGAATTCAAGCTTCT-3′	[24]
Tn*5* primer	5′-GGCCAGATCTGATCAAGAGA-3′	[24]
Psuppol-F	5′-ATAAACCAGCCAGCCGGAA-3′	This study
Psuppol-R	5′-TTCTGACAACGATCGGAGGA-3′	This study
BE5-F	5′-TCATGCAGGTGAATGTCGAT-3′	This study
BE5-R	5′-CTATCCGCAGGAGTTGAACG-3′	This study
BE53-F	5′-AGATTGATGTTGCCGAGGAC-3′	This study
BE53-R	5′-TGAAAAAGCTCCGTGAGGTC -3′	This study
BE85-F	5′-GCGCGGATATTGACATTGAT-3′	This study
BE85-R	5′-AGGCCGTCGATCTCTATCAC-3′	This study
BE103-F	5′-ACAAGAAGATGTCGGCCAAG-3′	This study
BE103-R	5′-TGCTCGCAGAATACAACTGC-3′	This study
BE168-F	5′-TCGAAAGCGCACTAGATTGA-3′	This study
BE168-R	5′-AGCCGTAAATATCGGACAGC-3′	This study
InnBXbaI-intF	5′-CGGTGGCGGCGGCCGCTCTAGAAAAATGCGCAACTGGAAGAT-3′	This study
InnBEcoRI-intR	5′-CGATAAGCTTGATATCGAATTCATCTGCTCACCAAGCCAATC-3′	This study

^a^ Pol^r^: Polymyxin resistant; Km^r^: Kanamycin resistant; Sm^r^: Streptomycin resistant; Sp^r^: Spectinomycin resistant; Tc^r^: Tetracycline resistant; Ap^r^: Ampicillin resistant; *inn*: incompatible nodulation. ^b^ United States Department of Agriculture, Beltsville, MD, USA.

**Table 2:** Results of BLAST and *tts* box searches of Tn*5*-flanking sequences.

		Gene with Tn*5* Insertion				
Strains	Nodulation on *Rj4* Soybean ^a^	Designated Symbol/GenBank Accession No. ^b^	Length (bp)	Deduced Gene Products	*tts* Box ^c^	Strain and Locus Tag/Gene (GenBank Accession No., % Identity by BLASTP Analysis)
BE5	+	*innA*/KX499540	1212	Cytosine deaminase	-	*Bradyrhizobium pachyrhizi* AOQ73_15990 (KRQ04415.1, 95%), *Afipia clevelandensis* ATCC 49720 HMPREF9696_04200 (EKS31979.1, 77%), *Agrobacterium rhizogenes* CN09_18315 (KEA04550.1, 61%), *Bradyrhizobium diazoefficiens* NK6 NK6_7958 (BAR61109.1, 59%), *Bradyrhizobium* sp. BTAi1 BBta_7204 (ABQ39084.1, 59%), *Nitrosospira* sp. NpAV SQ11_05400 (KIO49569.1, 58%), *Mesorhizobium loti* NZP2014 A8146_24615 (OBQ73313.1, 56%), *Azorhizobium caulinodans* ORS571 AZC_1945 (BAF87943.1, 49%), *Sinorhizobium meliloti* 1021 SMc02420 (CAC47175.1, 40%), *Pseudomonas fluorescens* C3 VC34_22545 (KJZ38903.1, 43%)
BE53	-	*innB*/KX499541	2280	Hypothetical protein	+	*Bradyrhizobium yuanmingense* BR3267 AOQ72_03805 (KRP85897.1, 73%), *B. japonicum* USDA6 BJ6T_78540 (BAL13100.1, 71%), *B. diazoefficiens* USDA110 blr1998 (BAC47263.1, 70%), *B. japonicum* USDA 6 BJ6T_78550 (BAL13101.1, 73%), *B. japonicum* USDA 6 BJ6T_78530 (BAL13099.1, 66%), *B. diazoefficiens* USDA110 bll1877 (BAC47142.1, 46%), *M. loti* MAFF303099 mlr6327 (BAB52639.1, 45%)
BE85	+	*innC*/KX499542	504	TerB family tellurite resistance protein	-	*Bradyrhizobium erythrophlei* MT12 SAMN05444164_5326 (SED64040.1, 97%), *B. diazoefficiens* NK6 NK6_3937 (BAR57108.1, 81%), *B. japonicum* Is-34 MA20_25200 (KGT77838.1, 80%), *B. japonicum* USDA6 BJ6T_05250 (BAL05822.1, 79%), *A. clevelandensis* ATCC 49720 HMPREF9696_03100 (EKS33980.1, 77%), *Proteobacteria bacterium* SG_bin9 A4S14_11410 (OQW55861.1, 76%), *Rhodopseudomonas* sp. AAP120 IP86_13420 (KPF97560.1, 73%), *Nitrobacter hamburgensis* X14 Nham_0605 (ABE61494.1, 72%)
BE103	+	*innD*/KX499543	1089	ABC transporter substrate-binding protein	-	*B. pachyrhizi* BR3262 AOQ73_18465 (KRQ01332.1, 99%), *B. diazoefficiens* USDA110 blr7816 (BAC53081.1, 87%), *Afipia* sp. P52-10 X566_18245 (ETR74761.1, 62%), *Rhodopseudomonas palustris* BisB5 RPD_2049 (ABE39284.1, 61%), *Azospirillum brasilense* Sp7 AMK58_19775 (ALJ37675.1, 61%), *B. japonicum* USDA6 BJ6T_61450 (BAL11399.1, 60%), *Bosea vaviloviae* SD260 AE618_12875 (KPH80633.1, 61%), *Agrobacterium tumefaciens* KCJ17 A7J57_24755 (OAE45812.1, 59%), *B. diazoefficiens* USDA110 blr3743 (BAC49008.1, 59%)
BE168	+	*innE*/KX499544	2292	GTP pyrophosphokinase	-	*B. pachyrhizi* BR3262 AOQ73_36790 (KRP86030.1, 99%), *Bradyrhizobium jicamae* PAC68 CQ12_18875(KRQ98494.1, 94%), *B. diazoefficiens* USDA110 bll5065 (BAC50330.1, 88%), *B. japonicum* USDA 6 BJ6T_46480 (BAL09914.1, 87%), *R. palustris* BisB18 RPC_2635 (ABD88185.1, 87%), *S. meliloti* 1021 SMc02659 (CAC45644.1, 60%), *A. tumefaciens* KCJ1736 A7J57_18100 (OAE38384.1, 60%), *A. brasilense* Sp7 AMK58_17855 (ALJ37338.1, 52%), *Frankia alni* ACN14a FRAAL2148 (CAJ60797.1, 40%)

^a^ Nodulation on *G. max* cv. BACR2 (*Rj4Rj4*) soybean; +: Positive; −: Negative. ^b^ *inn*: incompatible nodulation. ^c^ Presence of *tts* box in upstream regions of the genes; +: Present; −: Absent.

## Supplementary Materials

The following are available online at www.mdpi.com/2073-4425/8/12/374/s1. Figure S1: The location map of the identified genes *innA*, *innB*, *innC*, *innD*, and *innE* in *B. elkanii* USAD61 genome, Figure S2: Genome context of the identified genes *innA, innB, innC, innD*, and *innE*.
