# Supplementary material for: Identification of Bradyrhizobium elkanii Genes Involved in Incompatibility with Vigna radiata
Source: Genes (Basel). 2017 Dec 8;8(12):374. doi: 10.3390/genes8120374 (PMC5748692; doi:10.3390/genes8120374)
Supplement: Supplementary file 1 [file genes-08-00374-s001.pdf]

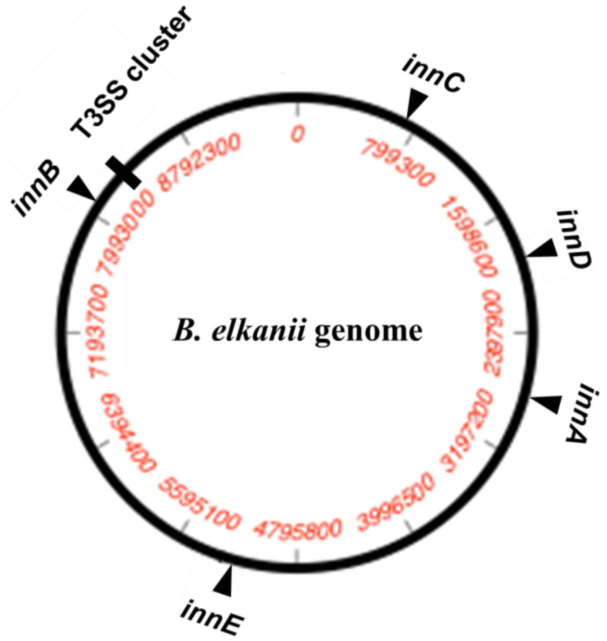

Fig. S1. The location map of the identified genes *innA*, *innB*, *innC*, *innD*, and *innE* in *B. elkanii* USAD61 genome. The location of T3SS cluster is indicated by the square box. The direction of the black arrows shows the location of the identified genes.

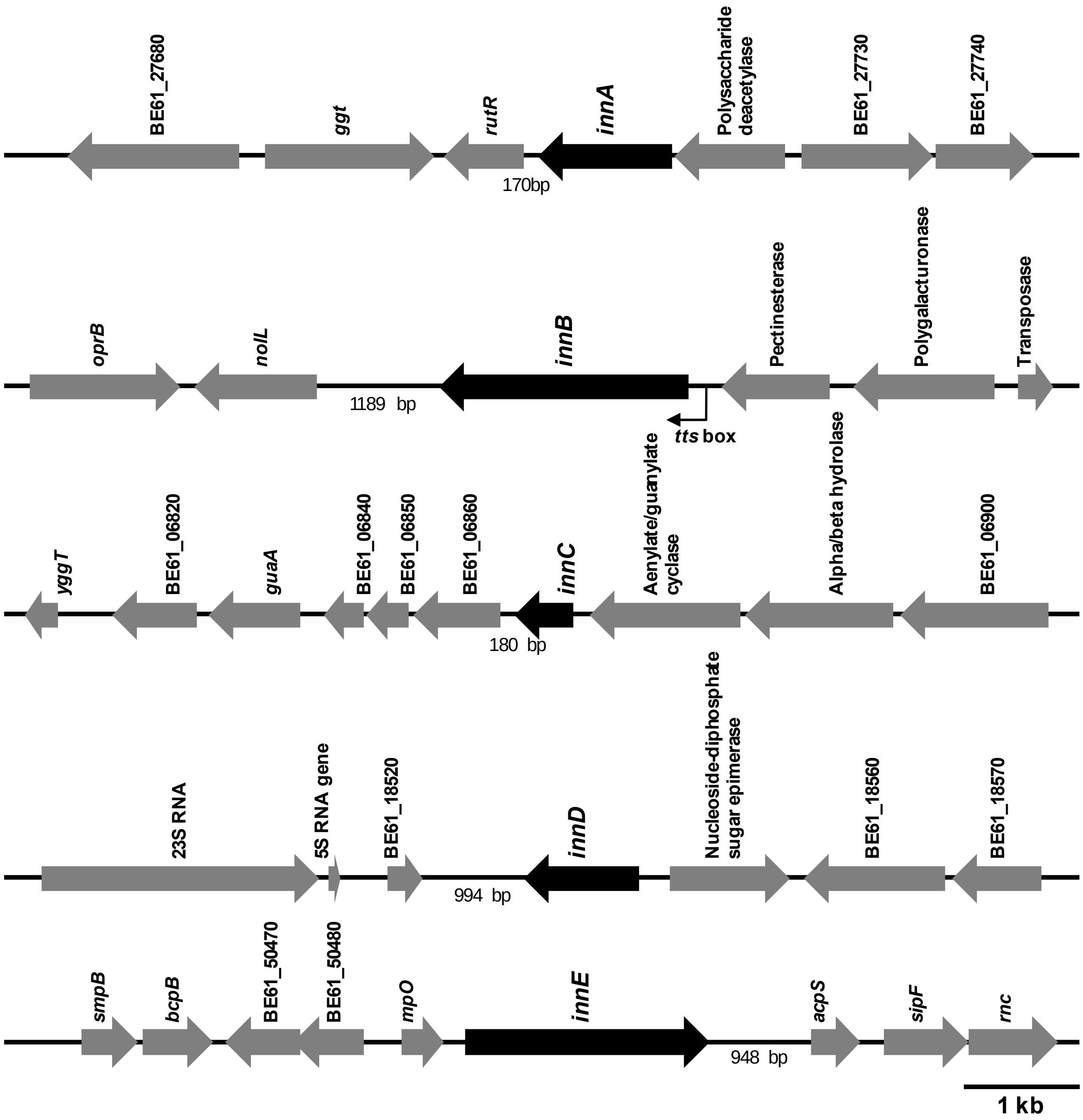

Fig. S2. Genome context of the identified genes *innA*, *innB*, *innC*, *innD*, and *innE*. Orientations and sizes of the predicted open reading frames (ORFs) are shown. The gene functions were estimated computationally for all predicted ORFs. The identified genes are shown by the black arrows. The putative *tts* box of *innB* is indicated by the black arrowhead. The number of base pairs between each *inn* gene and the next downstream gene is also shown.
